# Differences in seed characteristics, germination and seedling growth of *Suaeda salsa* grown in intertidal zone and on saline inland

**DOI:** 10.3389/fpls.2023.1175812

**Published:** 2023-10-24

**Authors:** Qikang Wang, Deliang Xu, Benfeng Yin, Yueling Zheng, Xiaohong Guo, Yating Li, Xiyan Sun, Lei Wang, Nan Wu

**Affiliations:** ^1^ School of Resources and Environmental Engineeringy, Ludong University, Yantai, China; ^2^ State Key Laboratory of Desert and Oasis Ecology, Xinjiang Institute of Ecology and Geography, Chinese Academy of Sciences, Urumqi, China; ^3^ School of Geography and Environment, Jiangxi Normal University, Nanchang, China; ^4^ Muping Coastal Environmental Research Station, Yantai Institute of Coastal Zone Research, Chinese Academy of Sciences, Yantai, China

**Keywords:** *Suaeda salsa*, seed exudate, salt stress, seed traits, seed germination

## Abstract

The ecological restoration of saline land in the Yellow River Delta is essential for the sustainability of this region. Halophytic species, like *Suaeda salsa*, are critical for the restoration process. However, potential differences in traits of heteromorphic seeds collected from the intertidal zone and inland condition have been largely overlooked. The seeds were analyzed for hardness, nutrient elements, and secretions, while structural differences were observed under a stereomicroscope. Germination percentages of the different seed types and subsequent seedling growth were also recorded. Our study found that the black seeds from intertidal zone had the highest hardness when compared to the three other types of seeds. Nutrient analysis revealed that brown seeds had a higher iron (Fe) content than black seeds. Accordingly, brown seed embryos were greener compared to their black seed counterparts due to the iron’s role in chlorophyll synthesis. Our results also revealed that brown seeds secreted greater amounts of exudates than black seeds. Finally, both the intertidal brown seeds and the inland-grown brown seeds had higher germination percentages and better early seedling growth than the corresponding black seeds. The differential characteristics between dimorphic seeds and seedlings may influence their environmental adaptation in different saline environments.

## Introduction

Seed germination and seedling establishment in halophytes, or salt-tolerant plants, are critical processes that determines the success of their survival and growth in saline environments. Halophytes are adapted to tolerate high salt concentrations (≥ 200 mmol NaCl) in their surrounding soil and water. However, the early stages of their life cycle, particularly seed germination and subsequent seedling establishment, can still pose challenges. These plants possess specific seed traits that allow them to tolerate salt stress. Environmental factors, especially salt concentration, play important roles in regulating seed germination, as well as the growth and survival of halophyte seedlings. Understanding the seed traits, germination process, and seedling establishment of halophytes is crucial for their conservation, restoration, and sustainable utilization in saline environments.

*Suaeda salsa* is a plant species belonging to the Amaranthaceae family (Liu et al., 2022). It was previously classified as an annual herb of the genus *Suaeda* in the Chenopodiaceae family and is commonly found growing in lakeshore wetlands and on beaches in Asia and Europe (Zhang et al., 2021). This species, a typical representative of halophytic plants, is able to grow under high salt concentrations and is widely distributed along the coastlines of China. Due to its ability to act as a biological barrier between coastal wetlands and the sea, *S. salsa* is considered one of the most effective nature-based solutions for shoreline stabilization and soil enhancement (Cao et al., 2022). Additionally, this species exhibits exceptional tolerance to salinity and flooding, and can accumulate large quantities of salt in its above-ground parts, making it a typical true salt plant (Wang et al., 2021). Furthermore, growing saline plants like *S. salsa* on saline soils using biological methods can significantly enhance and utilize such lands in the long term, reduce the area of saline land, and improve the ecological environment (Ao and Wu, 2019).

Indeed, a number of studies have investigated the tolerance and adaptation mechanisms of *S. salsa* to saline conditions. For example, Li et al. (2019) explored the effects of salinity on the germination, seedling emergence, seedling growth, and ion accumulation of *S. salsa* in the intertidal zone and on saline inland. Their results indicated that the salinity in the intertidal zone had a beneficial effect on the germination and seedling growth of *S. salsa*, but not on the saline inland. Additionally, Li et al. (2011) focused on the accumulation of ions during seed development under controlled saline conditions of two *S. salsa* populations. Their results suggested a positive correlation between the accumulation of potassium and sodium ions and the germination percentage and seedling growth of *S. salsa*, which may contribute to the species’ adaptation to saline environments.

Seed heteromorphism, or the existence of morphologically distinct seed types within a single plant species, has been widely observed among halophytes, such as *S. salsa* (Wang et al., 2015; Liu et al., 2018; Zhao et al., 2018). Seed heteromorphism is thought to facilitate plant establishment and survival in harsh environments by allowing for several distinct reproductive strategies that optimize the chances of persistence and dispersal. In *S. salsa*, two types of seeds have been described: black seeds, enclosed in a rigid seed coat, and brown seeds, covered by a soft seed coat. The two seed types differ in their germination requirements and percentages, with black seeds exhibiting higher dormancy and longer germination time than brown seeds (Wang et al., 2015; Zhang et al., 2021). These observations make *S. salsa* an interesting model species to explore the role of seed heteromorphism in plant adaptation to saline environments.

*Suaeda salsa* is a coastal halophyte plant that grows in both intertidal zones and saline inland areas. The different growth environments might cause variations in seed characteristics, seed germination percentage, and seedling growth, which could ultimately affect the survival and establishment of *S. salsa* populations. Therefore, this study aims to fill this gap by examining the following research questions: How do seed characteristics of *S. salsa* differ between the intertidal zone and saline inland? How does salinity influence the germination percentage and seedling growth of *S. salsa* in these two habitats? The answers to these questions will provide a better understanding of the adaptive mechanisms of *S. salsa* in different saline environments and facilitate the development of efficient strategies for conserving and restoring halophytes.

## Materials and methods

### Seed collection

In this experiment, freshly matured seeds of *S. salsa* were collected from two different locations, namely the intertidal zone (37.78°N, 119.17°E), and the saline inland (37.66°N, 118.93°E) of the Yellow River Delta in Shandong Province, China in October 2022. The seeds were stored dry at room temperature until they were used in these experiments.

### Seed traits

#### Thousand-grain weight

1000 seeds were randomly selected and weighed using the ME203E milligram balance produced by Mettler-Toledo Instrument (Shanghai) Co., Ltd. This process was repeated three times to ensure accuracy, and the resulting values were averaged to obtain the final measurement.

### Seed morphology

In order to visualize the seeds and embryos, the seeds were first soaked in distilled water for 12 hours and then peeled before being observed under a dissecting microscope (Stemi 508; Carl Zeiss Suzhou Co., Ltd.). Comparative analysis of the internal morphological structures of the seeds involved performing transverse sections and further examining the samples using scanning electron microscopy (SEM) with an S-4800 instrument.

### Seed hardness test

We selected 5 *S. salsa* seeds of similar size for each species, which were used as 5 repetitions. An electronic universal testing machine (C41.103; NSS(SHENZHEN) Laboratory Equipment Co., Ltd.) was utilized for compression testing. The compression was performed at a rate of 5mm/min using a 50 N sensor. The pressure was continuously increased until the stress-strain curve exhibited the first inflection point. This point represented the maximum pressure that the Saltbush seed coat could withstand.

### Seed secretions

#### Preparation

The preparation was conducted under controlled conditions of 25°C during the day and 20°C during the night. Each Petri dish, with a diameter of 3.5 cm, was seeded with 50 seeds, and 2ml of distilled water was added to each dish. Three replicates of each treatment were conducted for each type of seed and both habitats. After 48 hours of incubation, the secretions from the seeds were collected and subsequently centrifuged at 12000 rpm for 10 minutes at 4°C. The resulting supernatant was collected for further testing.

#### Soluble sugar and soluble protein

The quantification of these components was accomplished using the well-known anthrone method for measuring sugar content in biological samples. First, collect 1 ml of the secretions and centrifuge at 12000 rad for 10 min. Next, collect the resulting supernatant and kept for further use. Dilute the supernatant by a factor of 10. Then, sequentially added 0.1 mL of the diluted supernatant, 0.3 mL of distilled water, 0.12 mL of the working solution from the kit (Suzhou Geruisi Biotechnology Co., Ltd Suzhou, China), and 1 mL of H_2_SO_4_ to the EP tube. Mix the contents thoroughly. Cool the mixture to room temperature and read the absorbance at 620 nm using a UV-600T spectrophotometer. Finally, use the standard curve to determine the soluble sugar content.

The bicinchoninic acid (BCA) assay was employed to quantify the soluble protein content. Collect 1 ml of the secretions and centrifuge at 12000 rad for 10 min. Retrieve the supernatant and dilute it by a factor of 10. Subsequently, add 0.02ml of the diluted supernatant and 0.8ml of BCA solution from the kit (Suzhou Geruisi Biotechnology Co., Ltd Suzhou, China) to the EP tube. Mix thoroughly and read the absorbance at 562 nm after cooling to room temperature. Use the standard curve to determine the soluble sugar content (Gonzalez and Pignata, 1994; Walker, 2009; Lassouane et al., 2013).

#### Proline

The free proline levels in plants were determined using the acid ninhydrin method (Monreal et al., 2007). *S. salsa* seeds weighing 150 mg (fresh weight) were accurately measured using a precision balance (0.001 g). The seeds were then frozen in liquid nitrogen and ground into a fine powder. Next, 5 mL of 3% sulfosalicylic acid was added, and the mixture was extracted in a boiling water bath for 20 minutes. The extract was cooled, transferred to a 10 mL centrifuge tube, and centrifuged at 8000 g for 10 minutes. Subsequently, 2 mL of the resulting supernatant was transferred into a clean test tube, and 2 mL of glacial acetic acid along with 3 mL of ninhydrin color development solution were added. The solution was then heated in a boiling water bath for 1 hour. After cooling, 5 mL of toluene was added to the solution and vortexed for 20 seconds. The absorbance of the resulting supernatant was measured at 520 nm using a UV-Vis spectrophotometer (UV-600T).

### Nutrient elements

To determine the content of a target element in a plant sample, take either 0.4g of dry seeds or 1g of wet material (precise to 0.0001g) and place it in a 25ml beaker. Then, add 3.5 ml of nitric acid to the beaker, soak overnight, and heat the mixture on a hotplate at around 100°C until the solid sample is fully digested. Next, add 0.5ml of perchloric acid to the resulting mixture and continue heating at around 140°C until the white fumes stop. If the residue is not white, repeat the digestion process with nitric and perchloric acid. Finally, extract the sample with 7% hydrochloric acid and dilute to an appropriate volume. The content of the target element can then be determined using an inductively coupled plasma emission spectrometer (Prodigy-plus).

### Seed germination

A seed germination experiment was conducted using a smart light incubator (GTOP-500Y). The light conditions were set to a 12-hour cycle of light followed by 12 hours of darkness, while the temperature conditions were maintained at 25°C during the day and 20°C at night. For each treatment, three replicates of 50 seeds were incubated in 3.5 cm diameter Petri dishes on two layers of filter paper moistened with 2ml of distilled water. The germination experiment lasted for 20 days, with the germination standard being that the radicle extended by 1 mm. The seeds were checked every other day during the germination process.

### Seedling growth

The soil used for this experiment was a saline soil collected in December 2021 from the Yellow River Delta area (37.65N, 118.92E), Dongying City, Shandong Province, China. The total soil nitrogen content was 0.056%, alkaline hydrolysis nitrogen was 58.44 mg/kg, available phosphorus was 6 mg/kg, available potassium was 167.67 mg/kg, organic matter was 15.74 g/kg, pH was 8.3 and electrical conductivity value was 1841.34 μs/cm.

Different types of seeds were sown in seed starting trays filled with mentioned soil. Each dish (d=3.5 cm) contained 30 seeds of the same type. There were three dishes for each seed type collected from different habitats. The trays were incubated at a temperature of 25°C and subjected to a 12-hour light/12-hour dark cycle. After 25 days, thinning was carried out, and only 15 seedlings were retained per dish. However, if the number of seedlings was less than 15, no thinning was done. Plant height was measured every three days starting from the fourth day after the germination of the seedlings. On the 43rd day of growth, three plant seedlings were randomly selected, and the number of leaves they had was recorded.

### Statistical analyses

We conducted a one-way ANOVA on seed germination data and used the LSD method for multiple comparisons. We compared the differences in seed germination under different salt gradients (p < 0.05), and represented the data using multiple bar charts, with significant differences indicated by different letters. Additionally, we performed a one-way ANOVA on seed coat hardness, seed nutrient element, seed secretion, seedling height, and leaf number data. We created bar charts for seed coat hardness, seed nutrient element, seed secretion, and leaf number data, and a scatter plot for seedling height data. Lastly, we conducted a correlation analysis using seed nutrient element and secretion data to determine the relationship between these factors.

## Results

### Seed traits

#### Thousand-grain weight of seeds

The thousand-seed weight of the intertidal brown and black seeds was 1.159 g and 0.932 g, respectively. Meanwhile, the thousand-seed weight of the inland brown and black seeds was 0.838 g and 0.627 g, respectively.

### Seed morphology

**Figure 1:**
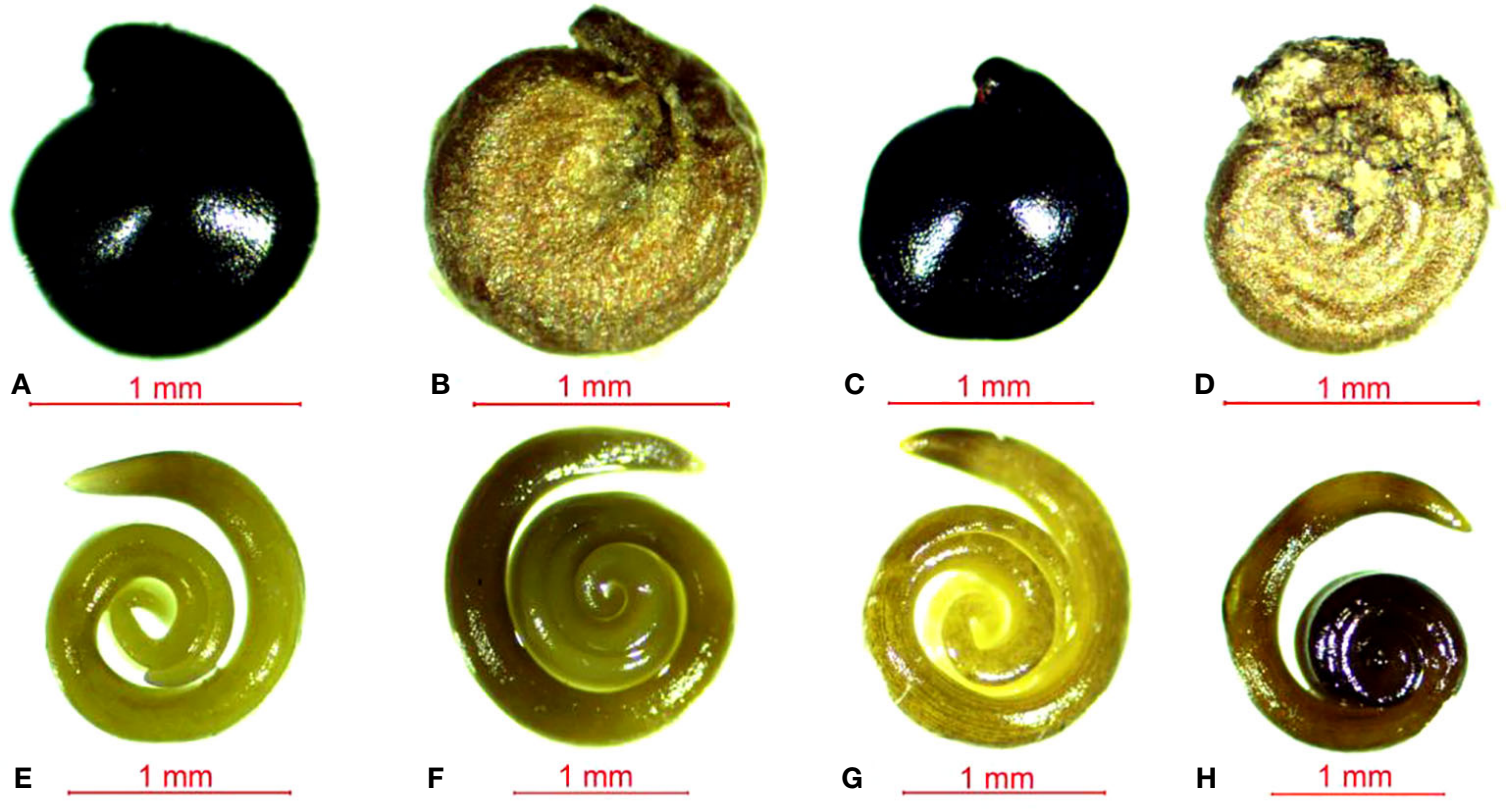
Seeds and embryos of *Suaeda salsa* under different growth states. **(A)** intertidal black seeds; **(B)** intertidal brown seeds; **(C)** inland black seeds; **(D)** inland brown seeds; **(E)** intertidal black seed embryos; **(F)** intertidal brown seed embryos; **(G)** inland black seed embryos; **(H)** inland brown seed embryos; the horizontal line indicates 1mm.

**Figure 2:**
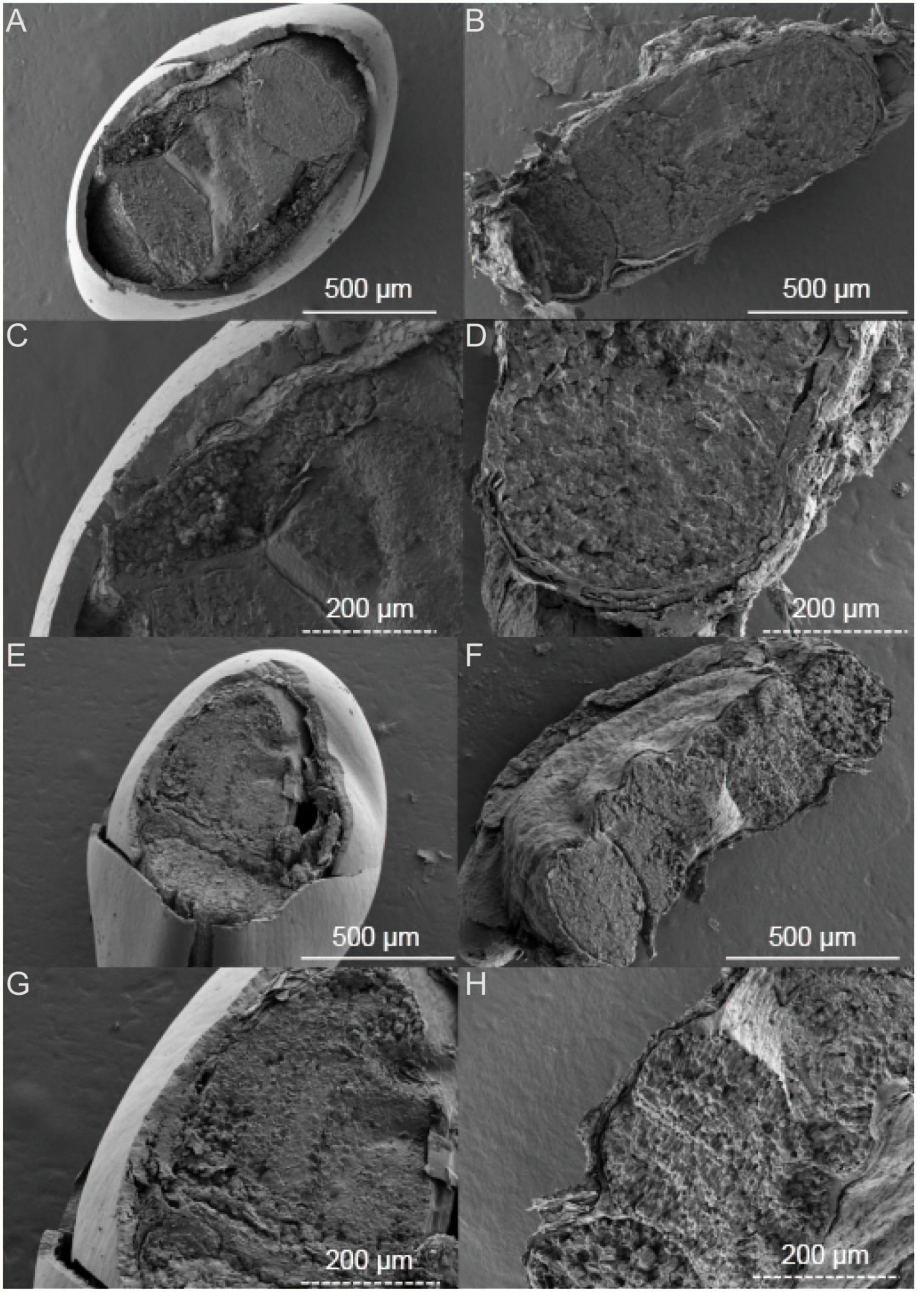
SEM images of *Suaeda salsa* under different growth states. **(A)** intertidal black seeds; **(B)** intertidal brown seeds; **(E)** inland black seeds; **(F)** inland brown seeds; **(C)** intertidal black seeds localized; **(D)** intertidal brown seeds localized; **(G)** inland black seeds localized; **(H)** inland brown seeds localized.

The black and brown seeds of *S. salsa* exhibit different physical characteristics. Specifically, the black seeds have a smooth epidermis and a hard shell while the brown seeds are flattened and have a loose epidermis. The intertidal black seeds are generally larger in size than inland-grown black seeds (**Figure 1**). Upon further observation of the embryo, we found that the color of the black seed embryo is lighter compared to that of the brown seeds. Additionally, the darker the seed coat, the lighter the embryo appears. In terms of seed structure, SEM images showcase that the black seeds have a tough outer testa, with the intertidal black seeds measuring around 50 μm in thickness and the inland black seeds measuring around 20 μm (**Figure 2**). Conversely, the brown seeds have a rectangular shape when viewed in cross-section and exhibit a loose epidermis with a distinct lamellar structure.

### Seed hardness test

**Figure 3:**
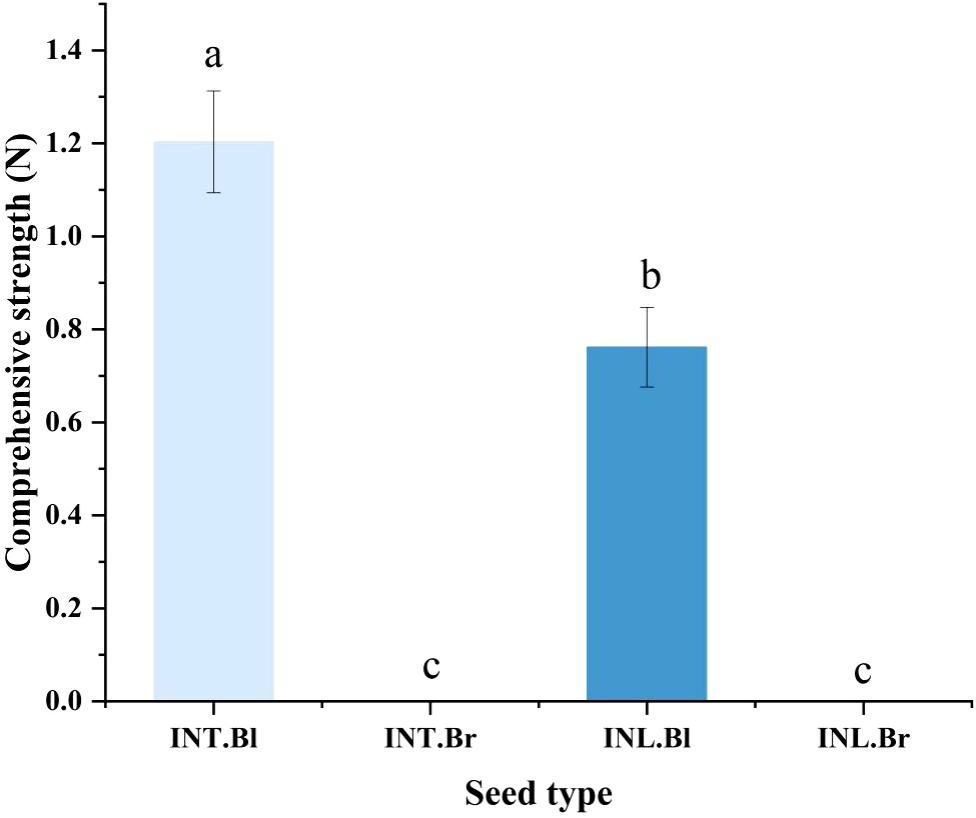
Comparison of seed coat hardness of *Suaeda salsa* under different growth states. INT. Bl, intertidal type black seeds; INT. Br, intertidal type brown seeds; INL. Bl, inland black seeds; INL. Br, inland brown seeds. Different lowercase letters indicate significant differences between species (P < 0.05).

We used a universal testing machine to examine the seed coat hardness of various varieties of *S. salsa* seeds, and ultimately discovered that the black seed coat of the intertidal type was substantially higher than the type produced inland (**Figure 3**).

### Soluble sugar and soluble protein

The brown seed exudate from the intertidal zone exhibited the highest soluble sugar content, reaching 372.472 μg/g. This value was significantly higher than the other three types of seeds (p < 0.05). Notably, the intertidal black termination and inland brown seed exudates showed minor differences in their soluble sugar content. In contrast, the inland black seed exudate had the lowest level of soluble sugar content.

**Figure 4:**
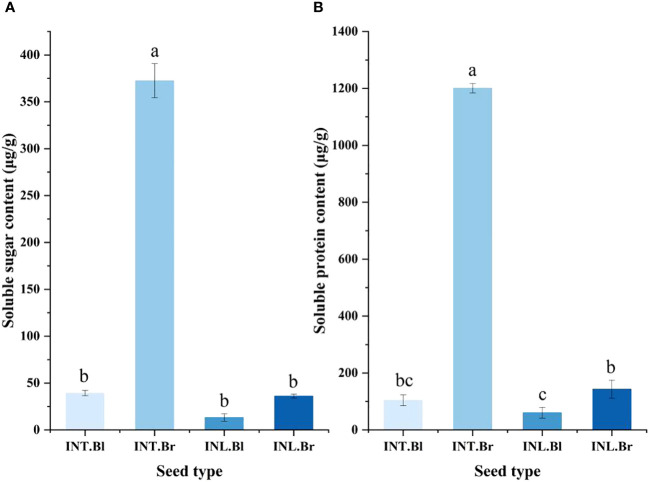
Effect of salinity treatment on Soluble sugar and Soluble protein content in seed secretion of *Suaeda salsa* under different growth states. **(A)**: Effect of salinity treatment on Soluble sugar content in seed secretion of *Suaeda salsa* under different growth states; **(B)**: Effect of salinity treatment on Soluble protein content in seed secretion of *Suaeda salsa* under different growth states. Different lowercase letters indicate significant differences between species (P < 0.05). INT. Bl, intertidal type black seeds; INT. Br, intertidal type brown seeds; INL. Bl, inland black seeds; INL. Br, inland brown seeds.

Furthermore, the results indicated that the soluble protein content in the seed secretions of *S. salsa* ponies differed depending on their growth state (**Figure 4**). Specifically, the intertidal brown seeds exhibited significantly higher levels of soluble protein content compared to the other three seeds (p < 0.05). The intertidal brown seeds also displayed the highest concentration of soluble protein content under the control treatment. However, there was no significant difference in the soluble protein content between the intertidal black seeds and the inland brown seeds. The lowest content was found in the grown inland black seeds, with a value of 50.67 μg/g.

### Proline

The proline content in the secretion of these four types of seeds did not differ significantly. The proline content in the secretion of these four types of *S. salsa* seeds did not differ significantly, and relatively the highest proline content of 31.19 μg/g was found in the secretion of inland brown, and the intertidal brown seed secretion the lowest proline content was 21.51 μg/g.

### Seed nutrient element analysis

Macronutrients, including K, Ca, S, P, Na, and Mg, were examined, revealing that intertidal black seeds had higher concentrations of Na, Ca, and S compared to planted black seeds. In contrast, intertidal brown seeds exhibited higher levels of S, K, Ca, Na, and Mg than planted brown seeds in different habitats. Moreover, black seeds within the same habitat had higher concentrations of Mg, Na, Ca, P, and K compared to brown-type seeds (**Table 1**).

**Table 1:** Changes of nutrient element concentration in seeds of *Suaeda salsa* in different habitats.

type of element (μg/g)	INT. Bl	INT. Br	INL. Bl	INL. Br
Cu	12.13 ± 1.13b	11.43 ± 0.32b	15.57 ± 0.07a	14.17 ± a
Mn	42.07 ± 5.72b	57.4 ± 3.8a	62.3 ± 0.57a	63.47 ± 1.84a
Zn	44.2 ± 5.96d	50.47 ± 1.4c	63.1 ± 0.1b	98.73 ± 1.7a
Fe	238 ± 46.11b	1988.33 ± 355.35a	148.33 ± 5.61b	503.33 ± 49.99b
B	11.68 ± 2.94a	19.83 ± 5.65a	15.06 ± 6.47a	13.08 ± 3.82a
Se	0.6 ± 0.14a	–	0.68 ± 0.09a	0.26 ± 0.1ab
Mg	2152.33 ± 29.46d	2625.33 ± 40.11a	2317.33 ± 14.34c	2430.67 ± 11.26b
Na	2806.67 ± 591.52b	6986.67 ± 533.74a	1571.33 ± 15.3b	1948 ± 81.75b
Ca	1703.67 ± 63.38c	10500.67 ± 1039.24a	1326.67 ± 21.53c	5585.33 ± 893.17b
P	5409.67 ± 549.66c	3589.33 ± 357.44d	7014.33 ± 23.55b	8582.33 ± 319.17a
K	7095.67 ± 498.05a	10608.33 ± 395.76a	7505 ± 38.43a	9662.33 ± 94.32a
S	2080.33 ± 197.38b	5837.33 ± 832.62a	1658.33 ± 82.5b	2816.67 ± 127.55b

The data are presented as the mean (n=3) ± standard error (S.E.), (–) indicates that it is not detected. Different letters indicate significant differences at different concentrations (p < 0.05). INT. Bl, intertidal type black seeds; INT. Br, intertidal type brown seeds; INL. Bl, inland black seeds; INL. Br, inland brown seeds.

The concentrations of Cu and Se elements were higher in black seeds than in brown seeds within the same habitat. Moreover, intertidal black seeds exhibited lower concentrations of Cu, Se, Mn, Zn, and B than black seeds grown inland under different habitats. Meanwhile, intertidal brown seeds had lower concentrations of Zn, Mn, Se, and Cu than brown seeds grown inland.

The nutrient concentrations in *S. salsa* seeds exhibited considerable variability, except for the B content. When comparing intertidal *S. salsa* with those grown inland, it was evident that the latter had notably higher levels of Cu, Mn, and P. On the other hand, *S. salsa* in the intertidal zone showed significantly elevated levels of Na, S, Ca, K, Mg, and Fe, whereas inland-grown *S. salsa* displayed markedly higher concentrations of Se. It is worth noting that inland-grown *S. salsa* also demonstrated significantly greater Zn concentrations compared to the other seeds.

### Germination

**Figure 5:**
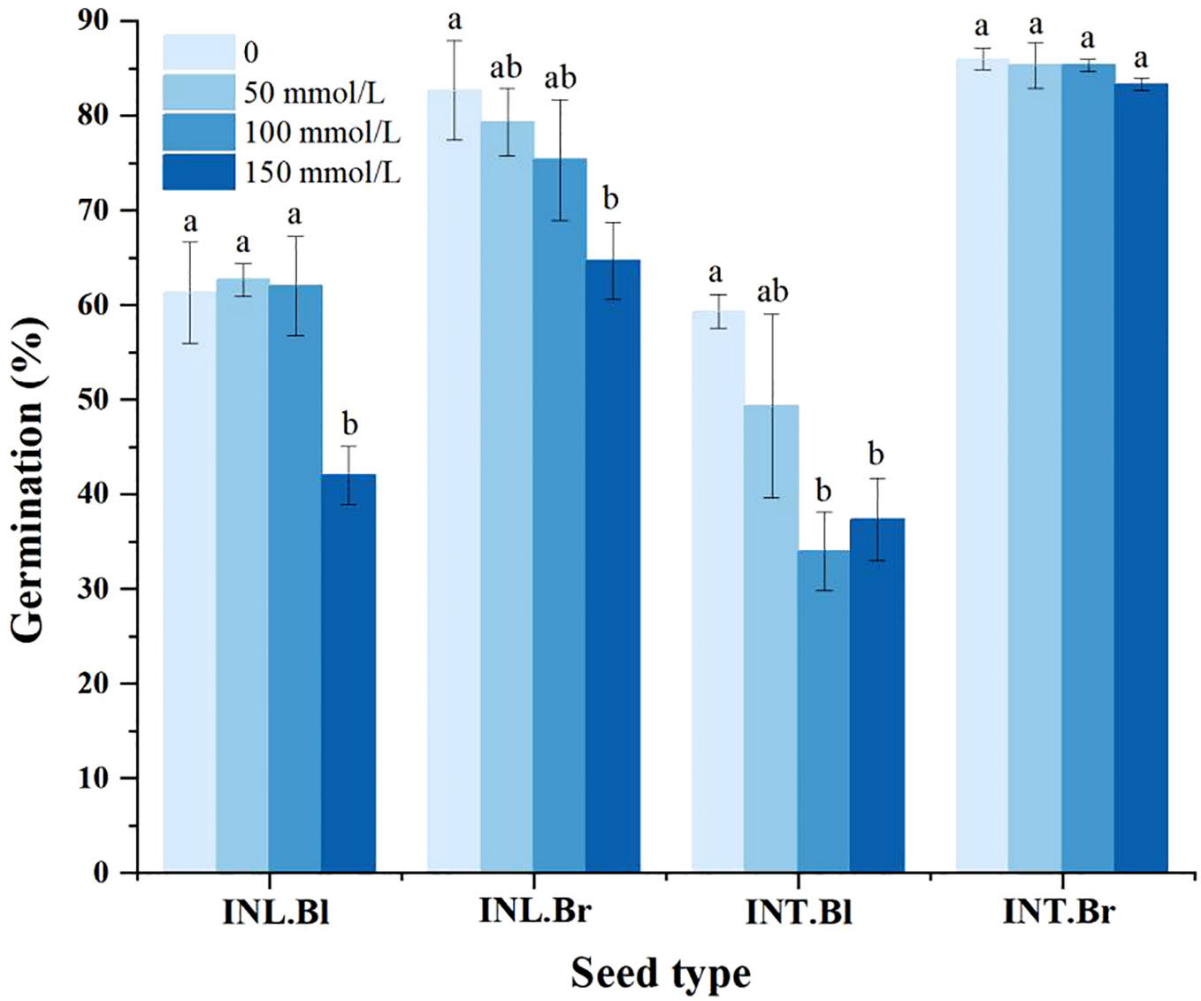
Germination percentage of *Suaeda salsa* seeds under different salt concentrations. Different lowercase letters indicate significant differences in the same species at different concentrations (P < 0.05). INT. Bl, intertidal type black seeds; INT. Br, intertidal type brown seeds; INL. Bl, inland black seeds; INL. Br, inland brown seeds.

The germination percentage of both intertidal and inland grown brown seeds was higher than that of corresponding black seeds at all salt concentrations (**Figure 5**). Compared to the control group, both intertidal and inland-grown brown seeds showed a decreasing trend in germination percentage. However, the decline was insignificant for intertidal brown seeds, while intertidal black seeds initially experienced a drop and then a partial recovery, but ultimately showed a significant decrease compared to the control group. Inland grown black seeds demonstrated fluctuations in germination percentage, showing a significant decrease under the 150 mmol/L treatment compared to the control. The germination percentage of seeds decreased with increasing salt concentration, indicating salinity’s inhibitory effect on the germination of both intertidal and inland seeds. Brown seeds were less affected than black seeds, and the inhibitory effect on intertidal brown seeds was negligible.

### Seedling characteristics

**Figure 6:**
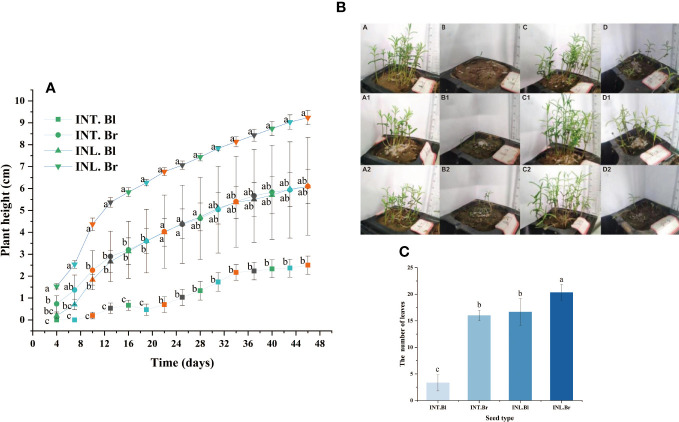
Seedling height of *Suaeda salsa* and number of seedling leaves of *Suaeda salsa* under different growth states. **(A)**: The graph was recorded from the 4th day after seed germination and every three days. Different letters indicate significant difference in plant height under different growth states on the same day (P < 0.05); **(B)**: Growth of *Suaeda salsa* seedlings. A, intertidal brown seedlings; B, intertidal black seedlings; C, inland brown seedlings; D, inland black seedlings. A1, the first replicate of the intertidal brown seedlings; A2, the second replicate of the intertidal brown seedlings; B1, the first replicate of the intertidal black seedlings; B2, the second replicate of the intertidal black seedlings; C1, the first replicate of the inland brown seedlings; C2, the second replicate of the inland brown seedlings; D1, the first replicate of the inland black seedlings; D2, the second replicate of the inland black seedlings; **(C)**: Number of seedling leaves of *Suaeda salsa* under different growth states. INT. Bl, intertidal type black seeds; INT. Br, intertidal type brown seeds; INL. Bl, inland black seeds; INL. Br, inland brown seeds. Different lowercase letters indicate significant differences between species (P < 0.05).

The plant height of brown seedlings grown inland exhibited the highest and fastest growth rate compared to other types. Similarly, the plant height of intertidal brown seedlings was greater than that of inland-grown black seedlings during the initial 17 days. However, beyond the 17th day, the growth rates were about the same, and the plant heights remained similar for both types. On the other hand, intertidal black seedlings had the lowest plant height, which was significantly different from the other three species. At day 43 of growth, three randomly selected seedlings from each type were analyzed, revealing that inland brown seedlings showed the highest number of leaves and better growth. While intertidal brown seed and inland-grown black seedlings had the same number of leaves, intertidal black seedlings had the lowest number of leaves and differed significantly from the others (**Figure 6**).

### Correlation analysis of seed traits

**Figure 7:**
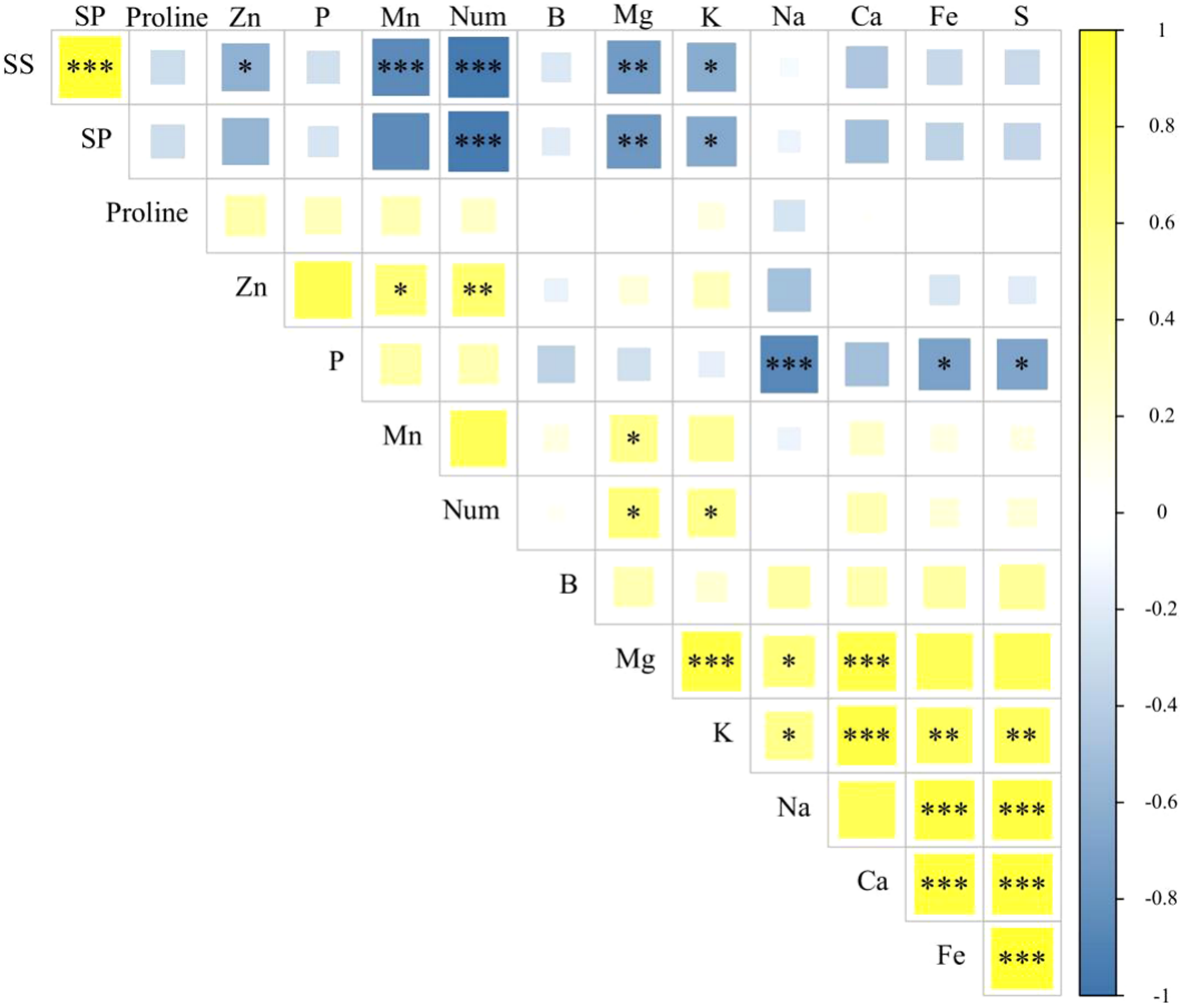
Correlation analysis of seed traits. SS, soluble sugar content; SP, soluble protein content; Num, the number of leaves at the 43rd day of growth of *Suaeda salsa* seedlings. *p < 0.05; **P < 0.01; ***P < 0.001.

There was a significant positive correlation (p < 0.01) between the Cu and P content in seed nutrients. Moreover, the Cu content showed a significant negative correlation (p < 0.05) with the content of soluble proteins. Additionally, Mn content had a significant positive correlation (p < 0.05) with the Zn and Mg content, as well as with the number of leaves (p < 0.01). However, it exhibited a significant negative correlation (p < 0.001) with the content of soluble sugars and soluble proteins. On the other hand, Zn content exhibited a significant positive correlation (p < 0.01) with the P content and leaf shape, but still showed a significant negative correlation (p < 0.05) with soluble sugars and soluble proteins (**Figure 7**).

Most of the nutrient elements were significantly positively correlated with each other. However, both the soluble sugar and soluble protein contents showed significant negative correlations with the nutrient elements. Remarkably, the number of leaves demonstrated a significant positive correlation with the nutrient content but exhibited a significant negative correlation with the soluble sugars and soluble proteins.

## Discussion

Although there has been extensive research on the seed traits and plant growth of *S. salsa*, this study is the first to document the characteristics of dimorphic seeds, germination, and seedling growth of *S. salsa* in both the intertidal zone and saline inland. Our findings clearly indicate notable distinctions between the two populations, suggesting that *S. salsa* has adapted to the environmental factors unique to each habitat. Additionally, our data reveals that dimorphic seeds from the same habitat also exhibit distinct differences.

Seed heteromorphism is a physiological and ecological mechanism developed by plants over time to adapt to challenging environments. This mechanism plays a crucial role in preventing overcrowding, reducing competition between offspring, and employing a bet-hedging strategy to cope with spatially and temporally diverse environments (Wang et al., 2010). For example, it has been demonstrated that the germination and survival of seedlings from heteromorphic seeds of *S. salsa* are successful under moderate stress from Zn and Cu metals (Zhang et al., 2020). Seed heteromorphism is the result of long-term adaptation of halophytes to a saline environment. Dimorphic seeds are common in Amaranthaceae. Large seeds have light color and exhibit no dormancy, while small seeds have dark color and deep dormancy (Wang et al., 2012; Cao et al., 2020). It is worth noting that black seeds have a relatively sturdy seed coat, especially the intertidal seeds, which have a tougher coating compared to the inland seeds. This characteristic provides excellent adaptation to external mechanical damage and equips them better for survive in harsher environmental conditions.

Soluble proteins and sugars are common osmoregulatory substances found in plants, and they play an important role in maintaining the osmotic pressure potential energy of cells, particularly under high salt stress conditions. Soluble sugars are crucial for osmoregulation during seed germination under salt stress. It has been observed that brown seeds accumulate more soluble sugars compared to black seeds. Song et al. (2022) found that *S. salsa* accumulates a variety of sugars such as sucrose, d-xylose, fructose, and glucose to help alleviate osmotic stress induced by prolonged exposure to salt environments. Moreover, the embryos of mature brown seeds contain higher levels of chlorophyll. It is noteworthy that the higher concentration of iron (Fe) in brown seeds compared to black seeds emphasizes its essential role in chlorophyll synthesis. This difference may be attributed to the higher activity of transporters in brown seeds, such as vacuolar Na^+^/H^+^ antiporter (NHX), potassium transporter (HAK), chloride channel protein (CLC) and Ca^2+^/H^+^ antiporter at tonoplast (CAX). These transporters are involved in maintaining ion homeostasis and improving water uptake for seeds during germination under salt stress (Liu et al., 2018). Furthermore, the results of this experiment revealed that the germination percentage of brown seeds was higher than that of black seeds under different salinity treatments. This finding is consistent with previous research, highlighting the significance of soluble sugars and iron in seed germination, particularly under adverse environmental conditions. Therefore, this study provides valuable insights into the mechanisms that determine successful seedling establishment (Venable, 1985; Tanveer and Shah, 2017).

Seed characteristics, such as seed mass, were significantly different between the intertidal and saline inland populations. Seed mass was found to be higher in the intertidal population, which may provide a better chance of survival in the harsh intertidal environment. Germination also varied significantly between the two populations. Seeds from the saline inland population had a higher germination percentage compared to those from the intertidal population. Generally, even under high salt stress, brown seeds can still germinate, but black seeds cannot germinate (Song et al., 2023). This may be due to the need for rapid seedling establishment and growth in the saline inland habitat. However, the germination percentage of the intertidal population was still high, indicating that the seeds have the potential to establish in both environments. Seedling growth of *S. salsa* was also significantly affected by the different habitats. Seedlings from the intertidal population had lower shoot length, root length, and biomass than those from the saline inland population (Liu et al., 2020; He et al., 2021). This may be due to the intertidal environment, which is characterized by a fluctuating water level and nutrient availability.

Soluble sugars play a crucial role in osmotic adjustment during seed germination under salt stress (Lin et al., 2013; Ortiz-Espín et al., 2017). Various factors can affect the stress tolerance of *S. salsa*, including differences in growing environments. Studies conducted by Zhou have shown that the content of soluble protein and soluble sugar in the roots and leaves of the same plant can differ across ecological zones, particularly in high tide lines (Zhou et al., 2015). Additionally, research by Peng has investigated differences in fresh weight, dry weight, photosynthetic pigments, and osmoregulatory substances such as organic acids, proline, and soluble sugars in the above-ground parts of *S. salsa* found in intertidal and saline habitats under varying salt concentrations (0, 200 mmol/L, 400 mmol/L) (Peng et al., 2017). Results showed that salt stress had a greater impact on *S. salsa* in saline habitats compared to intertidal habitats. Interestingly, all salt treatments caused an increase in the proline content in the seed secretion of *S. salsa*. Furthermore, seeds from intertidal zones had significantly higher soluble protein and soluble sugar content than those from inland zones. The cotyledons of large seeds develop more substantially than those of small seeds, such as *S. salsa* (Song et al., 2008). Fresh premature seed embryos contain chlorophyll and can undertake photosynthesis (Li et al., 2012). Consequently, the better developed embryos in brown seeds may provide more soluble sugar during germination, potentially accounting for the faster germination of brown seeds over black seeds in *S. salsa*.

One limitation of the study is that it does not investigate the genetic differences between the two populations. It is possible that genetic differences may also contribute to the observed differences in seed characteristics, germination, and seedling growth. Future studies could investigate the genetic differences between the two populations and how they relate to the observed differences in traits.

## Conclusion

The present study has shown clear differences between the intertidal and saline inland populations of *S. salsa* in terms of seed characteristics, germination, and seedling growth. These differences suggest that *S. salsa* has adapted to different environmental factors of the two habitats. The larger seed mass of the intertidal population may contribute to better seedling establishment and survival in the harsh intertidal environment. Additionally, the higher germination percentage of the saline inland population may be related to the need for rapid seedling establishment and growth in the more inconsistent soil environment.

## Data availability statement

The original contributions presented in the study are included in the article/supplementary material, further inquiries can be directed to the corresponding author/s.

## Author contributions

All authors listed have made a substantial, direct, and intellectual contribution to the work, and approved it for publication.
